# Visualizing and Understanding Shrinking Cities and Towns (SCT) Research: A Network Analysis

**DOI:** 10.3390/ijerph191811475

**Published:** 2022-09-12

**Authors:** Zezhou Wu, Danting Zhang, Shenghan Li, Jianbo Fei, Changhong Chen, Bin Tian, Maxwell Fordjour Antwi-Afari

**Affiliations:** 1 Key Laboratory for Resilient Infrastructures of Coastal Cities, Ministry of Education, Shenzhen University, Shenzhen 518060, China; 2 Sino-Australia Joint Research Centre in BIM and Smart Construction, Shenzhen University, Shenzhen 518060, China; 3 College of Civil and Transportation Engineering, Shenzhen University, Shenzhen 518060, China; 4 Gemdale Group South China Real Estate Company Shenhui Business Department, Futian District, Shenzhen 518026, China; 5 Department of Civil Engineering, College of Engineering and Physical Sciences, Aston University, Birmingham B4 7ET, UK

**Keywords:** urbanization, shrinking cities and towns, resilience, knowledge mapping, visualization

## Abstract

The world is undergoing an unprecedented trend of fast urbanization, which causes a range of socio-environmental consequences, one of which is shrinking cities and towns (SCT). SCT refer to the cities or towns that are experiencing population decline and economic downturn. In the existing literature, there have been numerous studies on SCT; however, there is a lack of study which investigates its knowledge domains. Therefore, this paper aims to conduct a scientometric analysis to achieve an outline of the SCT research status. Through the procedures of literature search and screening, a total of 716 SCT-related studies were extracted from the Scopus. The VOSviewer software system program was then utilized to visualize the present SCT-related studies. The visualization results revealed that the journal of Sustainability made significant contributions to the SCT research in terms of relevant publications. In addition, Haase, Annegret received the most co-citations, and was also the most productive author in this field. Furthermore, it was identified that current SCT research is mainly conducted in developed countries. Through the analysis of keywords, the emerging research topics were revealed. Discussions were further made from the perspectives of prevailing research methods, evaluation criteria, and solutions for SCT problems.

## 1. Introduction

Urbanization is one of the major social changes all over the world [1], which refers to the process of population gathering in urban areas or rural areas into urban areas. The fast speed of urbanization has caused a range of problems, such as global warming [2], construction and demolition waste production [3], the reduction in resident population [4,5,6] and exacerbated traffic congestion [7]. Shrinking cities and towns (SCT) are also a problem appearing in the fast urbanization process. In the literature, SCT have been defined as the phenomenon of the declining population and the gradual decline of economies in the cities with the process of deindustrialization [8,9,10]. It was not until 1988 that the term SCT was used to refer to hollowed-out inner cities where populations were largely lost under the influence of suburbanization [11]. There are many reasons for urban shrinkage, and the academic community generally summarized them into factors such as globalization [8,12], deindustrialization [13,14,15], suburbanization [16,17], demographic changes [18,19], changes in political context [20] and natural environment [21,22,23]. Although the performance of different SCT had certain similarities, the substantive reasons behind the phenomenon were not the same [24].

As the urbanization process continues to deepen, there are various factors affecting the process of urban development and recession [25,26,27]. The academic community has begun to use SCT to describe the worldwide urban transformation process and its impact. Wiechmann and Pallagst [28] provided an important summary of the evolution pathways and local policies of four SCT cases in developed countries. Barreira, et al. [29] assessed the residential satisfaction of four shrinking urban inhabitants in Portugal through a survey. It showed that shrinking caused by the deindustrialization is not conducive to the increase in satisfaction of citizens. Lang, et al. [30] examined how urban fringe villages are being transformed in the new agenda of urbanization in China. Although China is experiencing rapid urban expansion [31,32,33], China’s urbanization has begun to move toward stocks, and shrinkage is inevitable [34]. There is a need for an urban renewal approach to address and adopt additional development measures [35], for example, the method of optimizing the reverse logistics of construction waste proposed by Wu et al. [36]. Cortese, et al. [37] analyzed the efforts of three SCT cases to boost social coherence in the face of urban shrinkage, and examined issues that are driving aging and spatial inequalities in society. Haase, et al. [38] used an ABM approach to simulate residential mobilization in an SCT case. Currently, the research topic of SCT has been extensively studied. However, there are currently no studies that provide a comprehensive vision of the current SCT literature. Thus, this study endeavors to provide a scientometric review of the literature concerning SCT, and they do not fully reveal the connection between these studies. It also presents the holistic research status and evolutionary trends of SCT research.

The remainder of this paper is structured as follows. The following section describes the research methodology. Then, the documentary network is analyzed in terms of journals published, co-authors, countries, literature citations, and co-occurring keywords. A discussion is presented based on the results obtained. Finally, a Section 4 is included as an end.

## 2. Research Method

**Figure 1:**
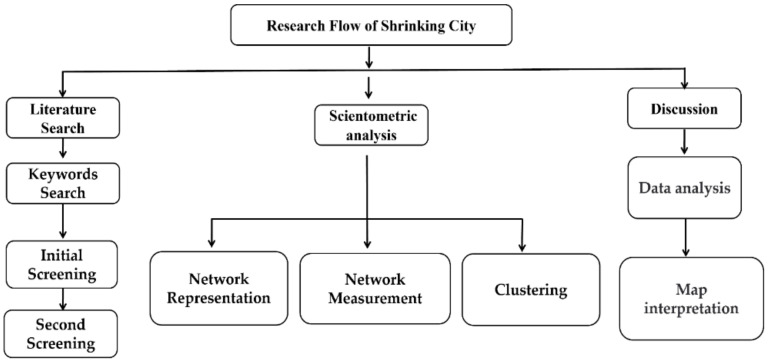
Research flow chart for reviewing the SCT literature.

This review-based study analyzed the SCT articles retrieved from the Scopus database which includes of the most prominent and influential journals in the world [39]. The overall flowchart is depicted in Figure 1, consisting of the literature collection, scientometric analysis, and discussions. After analysis and verification, the following search codes were implemented in the Scopus: TITLE-ABS-KEY (“shrinking city*” OR “shrinking town*” OR “urban decline” OR “city decline” OR “city shrinkage” OR “urban shrinkage”). Journal papers were targeted for this research, while book chapter, congress papers, books, reviews, editorials were excluded. This is due to the fact that journal articles generally contain more holistic and superior information than other kinds of publications [40,41].

**Figure 2:**
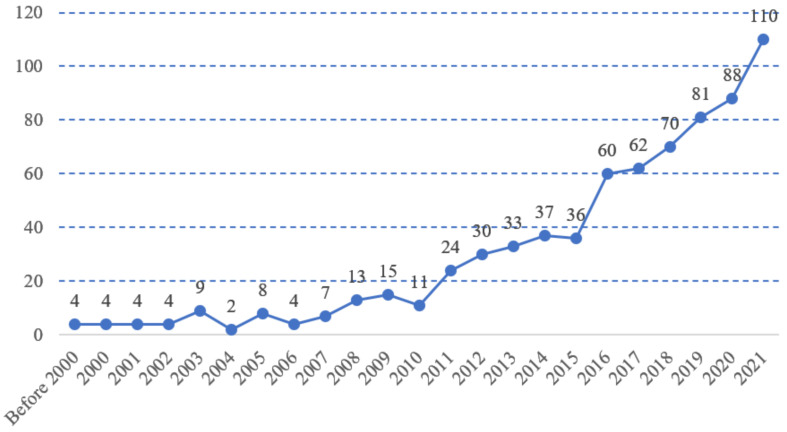
Line graph of SCT in Scopus in 2000–2021.

After a thorough search, a total of 716 bibliographical recordings were retrieved, ranging from 2000 to 2021, as shown in Figure 2. From Figure 2, it could be seen that the number of papers published before 2000 was limited; however, the trend in SCT research has been increasing since 2000. In particular, it has seen rapid growth since 2017. This indicates that SCT has gradually become a hot spot in urban economic and regional development research.

VOSviewer is one of the many scientific knowledge mapping programs, which realizes the mapping of scientific knowledge maps through the relationship construction and visual analysis of network data, and shows the structure, evolution and cooperation of knowledge fields [42,43]. As an open source tool with less obscurity in its installation and usage, its most distinctive feature is the ability to generate visual graphics [44]. In VOSviewer, the scale of a component is determined by the node. If colors have been specified for each item individually, each item’s circle is displayed in the color of the item [45].

Overall, this paper investigated the 712 journal articles which were published since 2000 in the field of SCT from five perspectives, including the number of publications, total citations, average citations, average normalized citations (ANC) and total connection strength. Since the five methods are intensely correlated, a synthesis of these five measures can serve to evaluate SCT-related studies. Average citation is a metric to evaluate the average impact for a journal or author, which is the reason normalization is needed to fix the misinterpretation that remoter articles gain more time to receive citations than more recent studies. ANC is equal to the total number of citations divided by the average number of citations published in the same year. Furthermore, it quantifies the result, which means a high score leads to a high impact as well [46]. The number of publications or total citations with the highest score may not have the highest ANC per article. The total connection strength indicates the scale of the node, referring to the total number of co-occurrences of keywords.

## 3. Results and Discussions

This section presents the mapping results of the derived SCT publication from five aspects, namely, journals, co-authors, countries, literature citations, and co-occurrence of keywords.

### 3.1. Mapping of Journals

**Figure 3:**
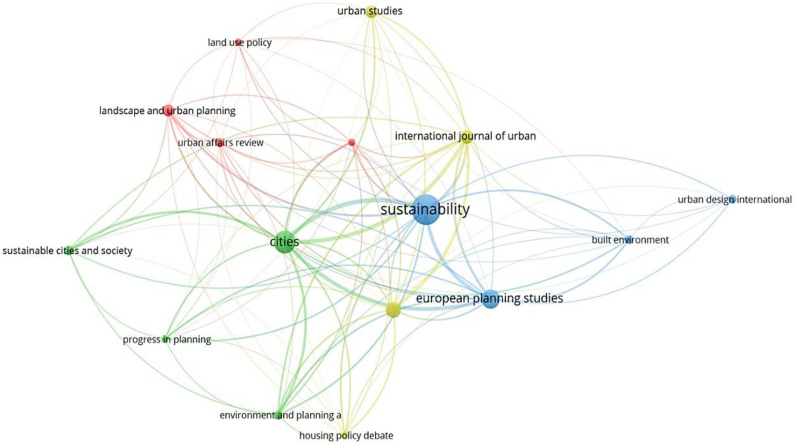
Map of dominant journals in the field of SCT.

In VOSviewer, the minimum number of published papers and the minimum number of citations are set to 5 and 113. A total of 16 out of 292 journals met this threshold. Figure 3 shown the clusters of journal provenance and the correlations by connecting lines.

It can be concluded that journals have devoted themselves substantially to the study of SCT as follows: Sustainability, Cities, European Planning Studies and Urban Geography. A more detailed description is given in Table 1.

In terms of the publications, Sustainability was the highest average productive journal. From this perspective of the total citations, Cities was cited the most times. In this respect of the average citations, Journal of the American Planning Association got the highest cited times. From the point of the ANC, Progress in Planning were the more influential journals on an annual basis. The results show that Progress in Planning has aroused great attention in SCT research which had a higher ANC score than others.

### 3.2. Co-Authors Analysis

**Figure 4:**
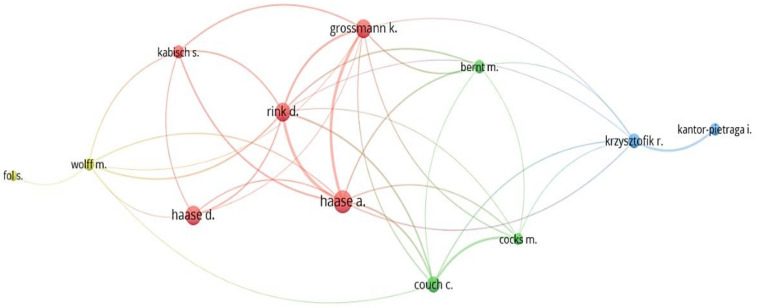
Co-authors analysis in SCT articles.

The authors of these articles can be obtained from bibliographic records, which identified scholars who have major research on SCT and academic collaboration between scholars [47]. The minimum of published articles set in VOSviewer is 5, and the minimum of citations for authors is 0, respectively. In total, 37 out of 1253 authors matched the selection criteria. Owing to the fact that several authors had no collaboration with others, there are a total of 12 authors displayed in Figure 4 and Table 2.

There were four different colors of red, blue, yellow and green, indicating that these authors were categorized into four groups of scholars in SCT. Haase, Annegret is the most prolific author, with 1179 total citations. The networks can further reveal the cooperative relationships of researchers within each network cluster. Others were dedicated to SCT-research considerably involving Haase, D, Grossmann, K, Rink, D, Couch, C, Krzysztofik, R, Bernt, M, Kabisch S, Kantor-Pietraga, I, Wolf, M, Cocks, M and Fol, S, who have been collaborative as visualized in Figure 4.

Table 2 indicates the information of emerging researchers involving Kantor-Pietraga, I, whose publications were generally around 2019. Bernt M, Haase, D, Rink, D and Grossmann, K have contributed significantly to the development of SCT. Table 2 also shows a very interesting phenomenon. Six of the authors have come from Helmholtz Center for Environmental Research, revealing that this institution plays a vital role in the research of SCT.

### 3.3. Countries Active in SCT Research

The minimum number of papers and citations per country is set to be 5 and 272. A sum of 12 out of 54 countries met the conditions.

**Figure 5:**
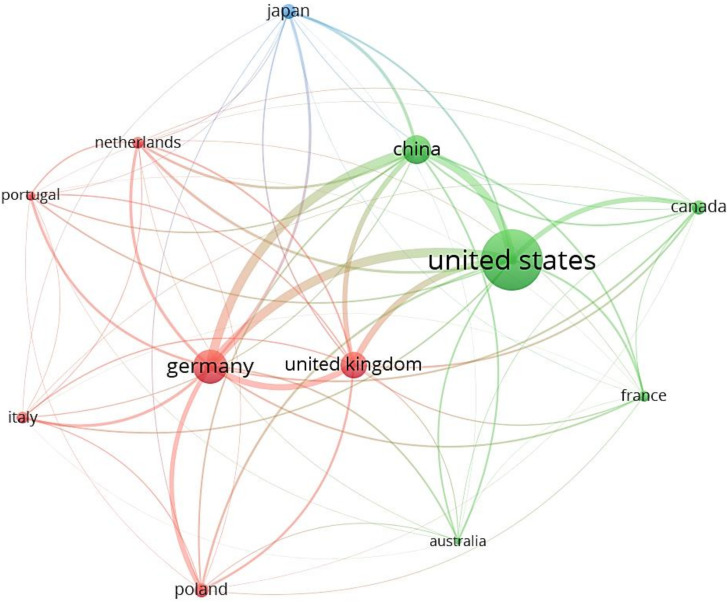
Mapping of countries active in SCT-related study.

The network connectivity model in Figure 5 shows the cross-references of the study between different countries. It is easy to find that the United States, Germany, China, the United Kingdom and Japan have become active in SCT research. In many countries, old industrial cities have made a series of planning and practical explorations to revitalize the city in response to challenges and transformation such as United Kingdom [18], the United States [16], China [48], and Germany [49].

A comprehensive analysis indicated a potential correlation between academic publications in a country and the influencing factors affecting urban shrinkage. However, the evidence articulating the relationship between these influences and country-specific academic outcomes is not clear. Research in this area needs to be explored. Details of the active countries were in Table 3.

The following countries have made significant commitments to SCT research: United States (243 articles), Germany (106 articles), China (82 articles), United Kingdom (72 articles), and Japan (32 articles), which implies that SCT research is developing faster in these countries. Scholars from the United States, Germany and China are in the first three positions in regard to the total citations, followed by the United Kingdom and Japan. The United States is at the central position and has the largest node, indicating that it has the greatest impact on SCT research. Other nodes such as Germany and China are also very large, and the connection strength with other countries is very close. ANC measurement indicates that these countries involving Australia, Germany, Netherlands, Canada, and China have created higher yearly impact. It is worth mentioning that most countries are developed countries and China is the only developing country in Figure 5. This indicates that urban shrinkage is becoming distinct in China.

### 3.4. Citation of Articles

**Figure 6:**
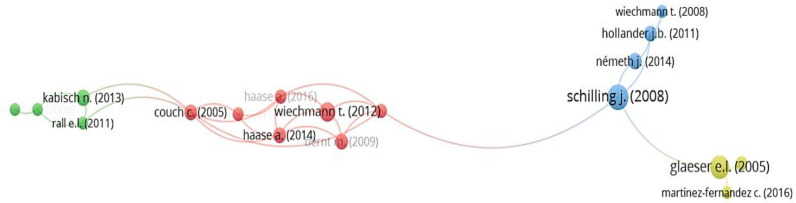
Scientific mapping of article impact in SCT-related study.

Article citations allow the underlying structure of a field of knowledge to be analyzed [46]. It also highlights the quality and recognition of the relevant references. The minimum citations per article was set at 112 and 18 articles were selected. The scientific mapping of article impact in SCT-related study is shown in Figure 6.

Schilling and Logan (2008) [50] received the highest number of citations in the past two decades, which presents a model for right sizing shrinking cities. Glaeser and Gyourko [51] received the second-highest citation rate. The third-highest-cited article offers an outline of the development paths and local strategies of four SCT cases: Dresden and Schwedt in eastern Germany; Pittsburgh and Youngstown in the US [28]. The article that received the highest ANC score was Haase et al. (2016) [52]. Details of the information cited in the articles are given in Table 4. Based on the high-cited articles listed, it can be concluded that, in addition to a general introduction to SCT, the existing applications comprise the causes of SCT [53,54], case comparison [28], theoretical study on SCT [55,56], and how to deal with the problems caused by SCT [50,57,58].

### 3.5. Co-Occurrence of Keywords

**Figure 7:**
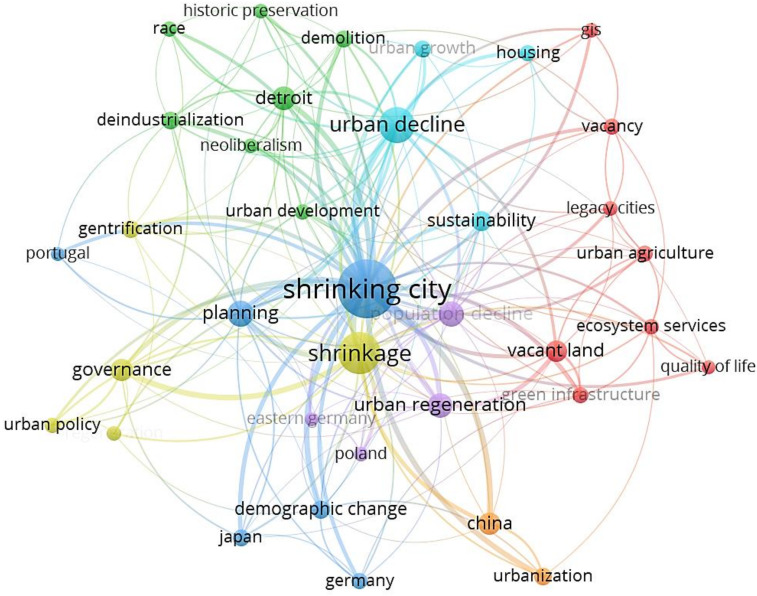
Co-occurrence of keywords in SCT research.

The word frequency analysis is a method of counting and analyzing the number of occurrences of important words in a document, and is an important tool for text mining. [66]. It corresponds to the “Author Keywords” and “Score Count” functions in VOSviewer [47]. The minimum frequency was set to 7. Ultimately, 36 out of 1823 keywords met this requirement as shown in Figure 7 and Table 5.

It can be found from Figure 7 that “shrinking city”, “demographic change” and “planning” are in the same cluster and associated intensely. Robust correlations also occur among keywords in different clusters, such as “shrinking city” and “urbanization”. On the whole, other keywords indicated that SCT were strongly associated with many factors, such as “demographic change”, “urban development”, “deindustrialization”, “governance”, “sustainability”, “vacancy”, “historic preservation”, “demolition”, and “urban regeneration”.

Table 5 summarizes detailed information of the keywords. According to the AC analysis, the following keywords including “green infrastructure”, “urban growth”, “vacant land”, “demolition”, and “governance” have received more attention. In those related articles, “Detroit” is the most typical shrinking city, and many scholars conducted in-depth studies on it. The Ave. Year Published shows the latest status of keywords in SCT research. For instance, research focusing on deindustrialization was extensively published in 2014, indicating that this topic has been studied early. On the contrary, papers related to urban regeneration, demographic change, urbanization were published around 2018, suggesting that these emerging subjects have attracted the attention of researchers in recent years. The values of ANC suggest that these words have received significant awareness, especially green infrastructure. The value is 2.0, which is higher than other keywords.

### 3.6. Discussions

The above analysis provided a holistic bibliometric review of the existing SCT research studies. Based on the derived results, a systematical analysis of the existing research was further discussed as follows.

(1) Case study acted as a key research method for the existing SCT studies. From the existing literature, it is interesting to find that case study is a commonly used research method in existing studies. In the early research stage, case study was employed to investigate the characteristics of a particular shrinking city or town. For example, Wiechmann [64] selected Dresden as a case to study the strategic adjustment to cope with population reduction. Lee, et al. [67] conducted a case study in Incheon to describe the built environment changes of shrinking cities. Such studies could provide valuable information to enhance the knowledge of SCT; however, due to the spatial limitation and timeliness of data, the research results may be hysteretic so as to affect its reference value.
(2) In the future, as the knowledge system of SCT needs to be broader, it could be foreseen that case study of a particular city or town will keep being a key research method in the field of SCT research. However, some other methods may also be proposed such as inductive analysis and data visualization method. The inductive analysis could explore the general laws and countermeasures of urban shrinkage, while the data visualization method provides a solid foundation for predicting, monitoring, and analyzing urban shrinkage.
(3) Population and economy are the two important indicators to evaluate SCT. So far, the quantitative standard to define an SCT has not reached a consensus. Some scholars defined SCT from the population perspective. For example, Oswalt, et al. [68] proposed that the urban population reduced as much as 10% reached a shrinkage. Meanwhile, some scholars used economic indicators to characterize SCT. For instance, Malamud [69] proposed to use the unemployment rate which is closely relevant to the overall economy of the city to represent the shrinkage of the city. Moreover, Alves, et al. [70] identified SCT by using demographic and economic changes in geospatial and scenic features.
(4) In the future, as SCT has swept throughout regions in both developed and developing countries, the evaluation criteria may be different by adding other indicators. For example, Schetke and Haase [71] believed that SCT could be calculated by parameters including the rate of building demolition and renovation. Bontje [72] proposed to use the vacancy rate to express urban shrinkage. The evaluation criteria should be determined considering the local economic and social situations.
(5) Finding solutions for the SCT problems has become the emphasis in SCT research. Despite the early studies on SCT characteristics, the recent research attempted to answer the questions of how to avoid and solve the problems of SCT. Local governments have formulated various strategies to deal with urban shrinkage. For example, Hospers [73] identified four strategies for issues involving urban governance and planning to respond to urban shrinkage. Farhan et al. [74,75] sorted out the historical changes of Najaf City, and committed to promoting and activating the protection of architectural heritage to realize the revival of the old city. Among the proposed strategies, effective planning is the dominant one to deal with urban shrinkage. It was recommended that planners should not only be responsible for “planning for growth”, but also be prepared to “plan for shrinkage”. For example, how to accurately predict the population size and population structure in the backdrop of SCT [76], and how to adjust the supply of urban infrastructure to adapt to the size and structure of the new population [77]. In addition, Hollander and Németh [56] proposed to adopt the mode of smart decline, that is, the city should be planned in a scientific, reasonable and appropriate way to avoid vicious competition between cities.

In the future, as the previous planning could not be changed, research on solving the SCT problems could focus more on urban renewal and regeneration. As claimed by Joo and Seo [78], to a certain extent, shrinkage is not the opposite of growth, but part of the expansion and austerity trend in the entire process of urbanization. Focusing on the renewal and utilization of the city’s stock space, SCT is an opportunity to prosper the city center. At a deeper level, SCT put new demands on the current urban government governance model. The government needs to rationally coordinate the relationship between the citizens and the land, and to reduce the waste of resources caused by the resistance to urban shrinkage. However, in order to realize city resilience, citizens were suggested to be extensively involved in the decision-making process concerning city revitalization.

## 4. Conclusions

SCT is an issue encountered during urban transformation and development, thus understanding its research status is essential for further enhancing relevant research. Through the network analysis, it was found that the early SCT research was mainly based on the causes, process, influence and the policy response of the government. In recent years, research has been gradually deepened and began to establish a comprehensive framework for SCT research. Furthermore, results show that Sustainability, Cities and European Planning Studies were the most influential publications in the SCT field. Research also demonstrated that Haase Annegret was identified with the highest average citation per individual publication. Developed countries have made great contributions to the study of SCT, such as the United States, Germany, and United Kingdom. The co-occurrence of keywords analysis identified the major clusters of SCT research, such as demographic change, land use change, deindustrialization, case comparison, urban environment, urban policy, urban planning, urban governance, and urban regeneration.

The research findings revealed in this study are useful for identifying the current research status and the key issues in SCT research. More importantly, it could help to highlight how the studies of SCT have evolved over time, greatly supporting the understanding of the basic structure of SCT research. At the same time, this study also guides the future research directions and puts forward higher requirements for urban planning. With the development of technology, more intelligent technologies, such as 5G technology, blockchain, and artificial intelligence, may be used to solve the technical problems in the process of urban shrinkage. However, this study also has some limitations. For instance, the data used in this study were based on the journal articles searched in Scopus; this may omit some relevant publications and studies in the form of conference papers and other types. The future studies could deal with such limitation by broadening the range of data source.

## Figures and Tables

**Table 1:** List of journal source in SCT-related studies.

Source	No. of Publications	Total Citations	Ave. Citations	Ave. Norm. Citations	Total Connection Strength
Sustainability	56	511	9	0.7	216
Cities	35	1044	30	2.0	264
European Planning Studies	26	923	36	1.3	172
Urban Geography	20	409	20	1.0	119
International Journal of Urban and Regional Research	15	971	65	1.8	175
Urban Studies	13	410	32	1.6	50
Landscape and Urban Planning	12	716	60	2.2	61
Sustainable Cities and Society	9	188	21	2.9	57
Environment and Planning a	8	404	51	2.3	99
Urban Affairs Review	8	213	27	1.2	33
Built Environment	7	191	27	0.6	55
Urban Design International	7	115	16	0.5	19
Housing Policy Debate	6	250	42	1.8	61
Journal of the American Planning Association	6	547	91	1.9	64
Land Use Policy	6	116	19	1.4	22
Progress in Planning	6	326	54	3.3	51

**Table 2:** List of productive scholars in SCT-related studies.

Scholar	No. of Articles	Total Citations	Ave. Year Published	Ave. Citation	Ave. Norm. Citations
Haase, Annegret	22	1183	2015	54	2.1
Haase, Dagmar	16	1212	2013	76	2.4
Grossmann, Katrin	15	870	2015	58	2.2
Rink, Dieter	15	1015	2014	68	2.3
Kabisch, Sigrun	8	272	2014	34	1.2
Wolff, Manuel	7	213	2018	30	2.1
Bernt, Matthias	8	678	2015	85	3.0
Couch, Chris	11	493	2013	45	1.8
Kantor-Pietraga, Iwona	7	75	2019	11	1.5
Krzysztofik, Robert	9	190	2018	21	1.5
Cocks, Matthew	6	271	2013	45	1.3
Fol, Sylvie	5	217	2015	43	1.7

**Table 3:** List of active countries in SCT-related studies.

Country	No. of Publications	No. of Citations	Ave. Year Published	Ave. Citation	Ave. Norm. Citations
United States	243	5050	2016	21	1.0
Germany	106	4011	2015	38	1.5
China	82	987	2019	12	1.3
United Kingdom	72	1968	2015	27	1.2
Japan	32	487	2016	15	1.3
Canada	31	825	2015	27	1.4
Poland	31	364	2018	12	1.0
Italy	24	433	2017	18	0.9
Netherlands	23	612	2015	27	1.4
France	22	482	2016	22	1.2
Portugal	17	309	2017	18	0.9
Australia	9	302	2017	34	2.3

**Table 4:** Details of articles citations in SCT.

Author	No. of Citations	Ave. Norm. Citations
Schilling and Logan (2008) [50]	437	5.4
Glaeser and Gyourko (2005) [51]	387	4
Wiechmann and Pallagst (2012) [28]	260	5.6
Kabisch and Haase (2013) [59]	190	6.3
Nemeth and Langhorst (2014) [60]	182	6.3
Haase et al. (2014) [24]	178	6.2
Hollander and Nemeth (2011) [56]	175	5.1
Bernt (2009) [57]	172	3.5
Couch et al. (2005) [61]	167	1.7
Grossmann et al. (2013) [58]	151	5
Haase et al. (2016) [52]	148	6.3
Rosenthal (2008) [55]	138	1.7
Reckien and Martinez-Fernandez (2011) [54]	130	3.8
Rieniets (2009) [53]	119	2.5
Rall and Haase (2011) [62]	117	3.4
Emmanuel and Kruger (2012) [63]	113	2.4
Wiechmann (2008) [64]	112	1.4
Martinez-Fernandez et al. (2016) [65]	112	4.8

**Table 5:** Summaries of main keywords in SCT.

Keywords	Occurrence	Ave. Year Published	Ave. Citation	Ave. Norm. Citations	Total Connection Strength
Shrinking City	241	2017	20	1.1	161
Shrinkage	104	2018	18	1.1	64
Urban Decline	72	2015	16	0.6	52
Planning	34	2017	14	0.9	32
Population Decline	31	2018	13	1.0	29
Urban Regeneration	29	2018	10	0.8	23
Detroit	26	2017	14	0.8	20
China	22	2019	15	1.9	17
Governance	22	2016	23	1.0	21
Vacant Land	20	2017	37	1.8	19
Sustainability	18	2017	16	0.7	12
Demographic Change	15	2018	17	1.0	14
Deindustrialization	13	2015	22	0.8	12
Gentrification	13	2017	20	0.9	10
Urbanization	13	2018	14	1.5	12
Demolition	12	2016	29	0.8	12
Japan	12	2018	17	1.7	11
Urban Growth	12	2016	37	1.6	9
Germany	11	2016	15	0.7	9
Green Infrastructure	11	2016	66	2.0	11

## Data Availability

The data presented in this study are available on request from the corresponding author.
